# Annual Meeting of the Association of Medical Officers of the Army and Navy of the Confederacy

**Published:** 1902-03

**Authors:** 


### Annual Meeting of the Association of Medical Officers of the Army and Navy of the Confederacy.

DALLAS, TEXAS, February 1, 1902.

The annual meeting of this association will be held in Dallas, Texas, April 22nd to 25th, inclusive, in connection with the annual meeting of the United Confederate Veterans. The committee of arrangements have sent out the following circular letter:

DALLAS, February 1, 1902.

DEAR DOCTOR: The Association of Medical Officers of the Army and Navy of the Confederacy will convene in Dallas, Texas, April 22nd to 25th, inclusive, during the meeting of the Confederate reunion. All surgeons, assistant surgeons, acting assistant surgeons, or contract physicians and hospital stewards, in the army and navy of the Confederate States, and all regular physicians who served honorably in any capacity in the Confederate States army and navy, and all regular physicians who are sons of Confederate veterans are eligible to membership.

You are cordially invited to attend said meeting and contribute reports of important cases coming under your observation, and any reminiscence worthy of preservation connected with your service in the army and navy of the Confederacy.

If you desire to become a member of the association and expect to attend the meeting next April, please write to the secretary, Dr. Blount, at once, giving time and place of enlistment; rank at time of enlistment; rank at close of war, character of service—army or navy; when and where surrendered, present address and remarks.

Any further information desired will be cheerfully furnished by Dr. E. A. Blount, or Dr. Deering J. Roberts, secretary of the association at Nashville, Tenn.

Respectfully,

HENRY A. MOSELEY, M. D.,
Chairman Local Executive Committee.

E. A. BLOUNT,
Secretary.

